# Nrf2在肿瘤化学防御方面的研究进展

**DOI:** 10.3779/j.issn.1009-3419.2011.07.10

**Published:** 2011-07-20

**Authors:** 惠琴 闫, 力 马, 清华 周

**Affiliations:** 1 300052 天津，天津医科大学总医院，天津市肺癌研究所，天津市肺癌转移与肿瘤微环境重点实验室 Tianjin Key Laboratory of Lung Cancer Metastasis and Tumor Microenviroment, Tianjin Lung Cancer Institute, Tianjin Medical Univercity General Hospital, Tianjin 300052, China; 2 300052 天津，天津医科大学总医院，肿瘤科 Department of Oncology, Tianjin Medical University General Hospital, Tianjin 300052, China

诱导正常细胞产生各种抗氧化酶、金属结合蛋白、药物代谢酶、药物转动蛋白及分子伴侣是防止肿瘤发生的有效措施之一。Nrf2（NF-E2-related factor 2）是肿瘤化学防御的一个重要细胞保护因子。许多细胞保护基因启动子区均有抗氧化反应元件ARE（antioxidant response element, ARE），Nrf2通过与细胞保护基因的ARE序列结合调节基因表达。正常条件下Nrf2位于细胞质，与氧化还原敏感性底物Keap1（Kelch-like ECH-associated protein 1）形成结合物并迅速通过泛素化途径降解。肿瘤化学诱导剂使Keap1巯基氧化后使Nrf2从Nrf2-Keap1结合物中释放并转入细胞核，诱导各种细胞保护基因表达。本文旨在对Nrf2转录因子近几年在肿瘤化学防御方面的研究进展加以综述。

## 1 肿瘤化学防御剂分类及作用

肿瘤的发生是多因素多阶段的复杂过程。通过诱导细胞保护蛋白调节致癌物质的代谢或分布是预防肿瘤的最有效措施之一。肿瘤化学防御剂可分为阻断剂（blocking agents）和抑制剂（suppressing agents）^[1]^。肿瘤化学防御剂可以是药物也可以是天然产品。其作用是防止正常细胞向肿瘤细胞的转化、抑制肿瘤细胞的启动或干扰前肿瘤细胞向恶性肿瘤细胞进展。一个好的肿瘤化学防御剂应该在正常细胞向肿瘤转化和启动阶段阻止各种内外界致癌物对DNA的损伤及修饰，停滞或逆转肿瘤的启动，在恶性肿瘤发生之前清除前恶性肿瘤细胞及相关有害物质。阻断剂的作用是抑制致癌物活化，增强已活化的致癌物的清除，诱捕其活性中间物的产生，加强DNA修复^[2]^。抑制剂的作用是抑制已转化的肿瘤细胞进一步恶化^[3]^。肿瘤化学防御剂可产生一定的活性氧族（reactive oxygen species, ROS）或亲电性物质，在细胞内产生一定程度的氧化应激^[4]^。这种轻微的氧化应激可有效地启动各种信号传导途径，诱导Ⅱ相解毒酶、Ⅲ相转运蛋白及各种抗氧化酶，从而激活抑癌基因的表达、抑制细胞增殖及血管生成^[5]^。研究发现多种肿瘤化学防御剂可活化Nrf2信号传导通路，通过转录因子Nrf2介导的抗氧化和抗炎作用防止肿瘤的发生。

## 2 Nrf2转录因子

Nrf2属于CNC-bZIP（cap’n’collar and basic leucine zipper）家族，位于染色体2q3.1，长约2.4 kb，是一个氧化还原敏感性的转录因子，富集于解毒反应最常发生的肠、肺和肾脏中^[6]^。通过Nrf2激活细胞的各种生存途径，参与抗氧化、抗炎、免疫反应、凋亡及肿瘤发生等多种生物进程^[7]^。Nrf2正性调节各种抗氧化剂、内外源性解毒酶和ATP依赖性耐药泵基因的表达^[8, 9]^，保护正常细胞免受氧化应激和各种内外源性有毒物质的损伤。Nrf2也可被MAPKs、PKC、PI3K及PERK等蛋白激酶磷酸化后激活，从Keap1结合物中解离转入细胞核调节各种靶基因的表达^[10]^。Nrf2-Keap1系统是机体氧化应激反应的一级防御体系^[11]^。正常条件下，Nrf2在胞浆内与抑制因子Keap1结合形成复合物，处于非活化状态。当内外源性毒性物质使Keap1的巯基氧化后，Nrf2从Nrf2-Keap1结合体中解离进入细胞核，与小Maf蛋白形成异二聚体识别ARE，表达目的基因，保护细胞特别是肺部组织细胞免受氧化损伤。Keap1决定Nrf2在细胞质和细胞核之间的穿梭与降解。Keap1缺失或变异导致Nrf2在细胞核中聚集，持续激活细胞保护基因的转录^[12]^。

## 3 Nrf2转录因子的调控

Nrf2是主要的ARE结合转录因子之一。各种肿瘤化学防御化合物高度依赖于Nrf2蛋白，通过激活Nrf2增加细胞的各种解毒酶及抗氧化酶活性，清除致癌物并阻止肿瘤的发生。有推测组成性表达Nrf2会使机体抵抗并耐受应激，维护机体健康，但事实并非如此。Nrf2的表达受各种因素的制约。它受体内外多种因素的诱导如过氧羟自由基、类胡萝卜素、醌类、联苯酚、迈克尔反应受体、异硫氰酸酯、重金属类、二硫化合物、血红素、NO、前列腺素和各种生长因子等因素的影响^[13]^。在正常生理条件下Nrf2与Keap1形成化合物迅速被泛素化并降解为26S蛋白酶。Keap1作为Nrf2和E3泛素连接酶的桥梁，导致Nrf2结构域中多个赖氨酸泛素化。Keap1使Nrf2转录因子泛素化依赖性的降解并可抑制Nrf2靶基因的表达^[14]^。Keap1既可以在细胞质内也可以在细胞核内将Nrf2降解^[15]^。这可能是防止氧化应激时Keap1依赖性地将Nrf2在细胞质或细胞核内降解，导致Nrf2在核内的聚集。核内聚集的或稳定的Nrf2方可与ARE序列结合，进而与Maf小蛋白分子结合启动Nrf2靶基因的表达。

## 4 Keap1对Nrf2的调控

Keap1位于染色体19p13.2上，长2.6 kb。Keap1决定Nrf2在细胞质细胞核之间的穿梭与降解。Keap1缺失导致Nrf2在细胞核中组成性地聚集，激活细胞保护基因的转录。Keap1在正常情况下通过泛素26S溶酶体途径调节Nrf2降解。最近的研究^[16]^表明Keap1靶向作用于Cul3的E3连接酶使Nrf2泛素化。Keap1通过防止Nrf2在核中聚集并增加溶酶体降解速率负性调节Nrf2。Keap1是一个富含半胱氨酸的蛋白，其中的27个半胱氨酸残基作为感受器可探测氧化剂和外源性物质。C257、C273、C288和C297残基可与Nrf2的N未端的Neh2结构域相互作用。其中C273和C288的变异会消除Keap1对Nrf2的抑制作用^[17]^。有研究^[18]^表明Cys151点突变为Ser不可逆地抑制Nrf2，用氧化应激因子处理后低表达或不表达ARE驱动的基因。而在Keap1^-/-^小鼠体内敲入Keap1C151S则恢复ARE驱动的基因的表达^[19]^。值得注意的是在体内或体外Keap1的活性半胱氨酸都可被外源性的亲电性物质所修饰。Keap1作为氧化应激敏感性的分子开关，负性调控Nrf2靶基因的表达。

## 5 Nrf2调控的靶基因

通过转录因子Nrf2可诱导药物代谢酶和抗氧化蛋白。Nrf2化学诱导剂的主要作用在于调节各种致癌物的吸收、分布、代谢、排泄及抗炎反应。慢性炎症反应是肿瘤发生的一个危险因子，肿瘤化学防御剂的作用其中就包括Nrf2的抗炎反应。Nrf2-Keap1系统的靶基因主要有4个方面^[13]^，包括①Ⅱ相解毒酶，如：谷胱甘肽硫转化酶（glutathione S-transferases, GSTs）、醌氧化还原酶（quinone oxidoreductase, NAD(P)H）、UDP-葡萄糖醛酸转移酶1A6（UDP-glucuronyl transferase 1A6）、黄曲霉毒素B1醛还原酶（aflatoxin B1 aldehyde reductase）及微粒体环氧化物水解酶（microsomal epoxide hydrolase）；②抗氧化蛋白，如血红素氧化酶（hemeoxygenase-1, HO-1）、过氧化酶1（peroxiredoxin 1）、谷胱甘肽过氧化物酶（glutathione peroxidase）、过氧化物岐化酶（superoxide dismutase）及硫氧还蛋白（thioredoxin）等；③GSH生成酶，如γ-谷氨酰半胱氨酸合成酶（γ-glutamylcysteine synthetase, γ-GCS）调节并维持细胞内GSH水平；④ Ⅲ相转运蛋白。

## 6 诱导Nrf2的肿瘤化学防御剂

Nrf2转录因子的主要诱导剂有类胡罗卜素、异硫氰酸酯、砷衍生物、迈克尔反应受体、前列腺素及各种生长因子等^[13]^，同时也包括诱导其靶基因血红素氧化酶的一系列肿瘤化学防御剂如姜黄素（curcumin）、莱菔硫烷（sulforaphane）、鼠尾草酚（carnosol）及白藜芦醇（resveratrol）等化合物^[20]^，这些化合物均通过活化转录因子Nrf2诱导*HO-1*基因及蛋白的表达。细胞的生存取决于细胞或组织对应激的耐受、修复及对损伤分子或转化细胞的清除。对正常细胞而言活化Nrf2诱导HO-1的表达起到防御肿瘤发生的作用。然而有研究^[21]^报道与癌旁组织相比，肺癌、腺癌、黑色素瘤、胰腺癌及脑瘤等多种肿瘤组织中HO-1为高表达。HO-1及其代谢产物具有抗氧化、抗炎、抗凋亡、促血管生成及促肿瘤侵袭转移的作用。表明HO-1既能保护正常细胞向肿瘤细胞转化，又能保护肿瘤细胞在恶劣的氧化应激环境中生存。对肿瘤细胞而言诱导HO-1表达会促进肿瘤的生长及恶化，提示肿瘤化学防御药物应用应谨慎，肿瘤化学防御药物并非都具有抑制肿瘤生长及恶化的作用。

## References

[1] Watenberg LW (1985). Chemoprevention of cancer. Cancer Res.

[2] Watenberg LW (1993). Prevention-therapy-basic science and the resolution of the cancer problem. Cancer Res.

[3] Hu R, Saw CL, Yu R (2010). Regulation of NF-E2-related factor 2 signaling for cancer chemoprevention: antioxidant coupled with antiinflammatory. Antioxid Redox Signal.

[4] Paolini M, Perocco P, Canistro D (2004). Induction of cytochrome P450, generation of oxidative stress and *in vitro* cell-transforming and DNA damaging activities by glucoraphanin, the ioprecursor of the chemopreventive agent sulforaphane found in broccoli. Carcinogenesis.

[5] Hu R, Kong AN (2004). Activation of MAP kinases, apoptosis and nutrigenomics of gene expression elicited by dietary cancer-prevention compounds. Nutrition.

[6] Motohashi H, O'Connor T, Katsuoka F (2002). Integration and diversity of the regulatory network composed of Maf and CNC families of transcription factors. Gene.

[7] Cho HY, Reddy SP, Kleeberger SR (2006). Nrf2 defends the lung from oxidative stress. Antioxid Redox Signal.

[8] Hayashi A, Suzuki H, Itoh K (2003). Transcription factor Nrf2 is required for the constitutive and inducible expression of multidrug resistance-associated protein 1 in mouse embryo fibroblasts. Biochem Biophys Res Commun.

[9] Vollrath V, Wielandt AM, Iruretagoyena M (2006). Role of Nrf2 in the regulation of the Mrp2 (ABCC2) gene. Biochem J.

[10] Jeong WS, Jun M, Kong AN (2006). Nrf2: A potential molecular target for cancer chemoprevention by natural compounds. Antioxid Redox Signal.

[11] Li N, Nel AE (2006). Role of the Nrf2-mediated signaling pathway as a negative regulator of inflammation: implications for the impact of particulate pollutants on asthma. Antioxid Redox Signal.

[12] Kwak MK, Kensler TW (2010). Targeting NRF2 signaling for cancer chemoprevention. Toxicol Appl Pharmacol.

[13] Kobayashi M, Yamamoto M (2005). Molecular mechanisms activating the Nrf2- Keap1 pathway of antioxidant gene regulation. Antioxid Redox Signal.

[14] Kaspar JW, Niture SK, Jaiswal AK (2009). Nrf2: INrf2 (Keap1) signaling in oxidative stress. Free Radic Biol Med.

[15] Nguyen T, Nioi P, Pickett CB (2009). The Nrf2-antioxidant response element signaling pathway and its activation by oxidative stress. J Biol Chem.

[16] Cullinan SB, Gordan JD, Jin J (2004). The Keap1-BTB protein is an adaptor that bridges Nrf2 to a cul3-based E3 ligase:oxidative stress sensing by a cul3- Keap1 ligase. Mol Cell Biol.

[17] Wakabayashi N, Dinkova-Kostova AT, Holtzclaw WD (2004). Protection against electrophile and oxidant stress by induction of the phase 2 response: fate of cysteines of the Keap1 sensor modifed by inducers. Proc Natl Acad Sci USA.

[18] Zhang DD, Hannink M (2003). Distinct cysteine residues in Keap1 are required for Keap1-dependent ubiquitination of Nrf2 and for stabilization of Nrf2 by chemopreventive agents and oxidative stress. Mol Cell Biol.

[19] Yamamoto T, Suzuki T, Kobayashi A (2008). Physiological signifcance of reactive cysteine residues of Keap1 in determining Nrf2 activity. Mol Cell Biol.

[20] Lou XL, Zhou XX, Liu Z (2008). Advance in the study of compounds inducing the expression of heme oxygenase-1. Yao Xue Xue Bao.

[21] Jozkowicz A, Was H, Dulak J (2007). Heme oxygenase-1 in tumors: is it a false friend?. Antioxid Redox Signal.

